# Attitudes towards terminal sedation: an empirical survey among experts in the field of medical ethics

**DOI:** 10.1186/1472-684X-6-4

**Published:** 2007-04-16

**Authors:** Alfred Simon, Magdalene Kar, José Hinz, Dietmar Beck

**Affiliations:** 1 Academy for Ethics in Medicine, Georg-August-University, Goettingen, Germany; 2 Department of Medical Ethics and History of Medicine, Georg-August-University, Goettingen, Germany; 3 Department of Anaesthesiology, Emergency and Intensive Care Medicine, Georg-August-University, Goettingen, Germany

## Abstract

**Background:**

"Terminal sedation" regarded as the use of sedation in (pre-)terminal patients with treatment-refractory symptoms is controversially discussed not only within palliative medicine. While supporters consider terminal sedation as an indispensable palliative medical treatment option, opponents disapprove of it as "slow euthanasia". Against this background, we interviewed medical ethics experts by questionnaire on the term and the moral acceptance of terminal sedation in order to find out how they think about this topic. We were especially interested in whether experts with a professional medical and nursing background think differently about the topic than experts without this background.

**Methods:**

The survey was carried out by questionnaire; beside the provided answering options free text comments were possible. As test persons we chose the 477 members of the German Academy for Ethics in Medicine, an interdisciplinary society for medical ethics.

**Results:**

281 completed questionnaires were returned (response rate = 59%). The majority of persons without medical background regarded "terminal sedation" as an intentional elimination of consciousness until the patient's death occurs; persons with a medical background generally had a broader understanding of the term, including light or intermittent forms of sedation. 98% of the respondents regarded terminal sedation in dying patients with treatment-refractory physical symptoms as acceptable. Situations in which the dying process has not yet started, in which untreatable mental symptoms are the indication for terminal sedation or in which life-sustaining measures are withdrawn during sedation were evaluated as morally difficult.

**Conclusion:**

The survey reveals a great need for research and discussion on the medical indication as well as on the moral evaluation of terminal sedation. Prerequisite for this is a more precise terminology which describes the circumstances of the sedation.

## Background

According to the definition of the World Health Organization (WHO) palliative medicine aims to enhance quality of life in patients suffering from an incurable disease [1]. When dealing with treatment-refractory symptoms it is a possibility to sedate patients in order to obtain satisfactory symptom control. In 1991, the use of sedation in imminently dying patients was called "terminal sedation" [2] for the first time and has since been controversially discussed not only within palliative medicine. While supporters regard terminal sedation as an indispensable palliative medical treatment option, opponents disapprove of it as "slow euthanasia" [3,4].

To date, only a few valid empirical data on the use of sedation to treat refractory symptoms in terminally ill patients are available. From the Netherlands it is known that terminal sedation is used in up to 10% of dying patients [5,6]. A study in six European Countries (Belgium, Denmark, Italy, The Netherlands, Sweden and Switzerland) revealed that continuous deep sedation was applied in 2.5% in Denmark and up to 8.5% in Italy. Of all patients receiving this kind of sedation, 35% (Italy) and up to 64% (Denmark and The Netherlands) did not receive artificial nutrition or hydration [7]. As regards other countries (including Germany), there are only figures of individual palliative care units [8-12]. The reasons for why they partly differ considerably may be that there is still no generally acknowledged definition and that the scope of how the term is used varies [13]. In the literature, one can also find alternative terms like "palliative sedation" [10,14,15] or "sedation at the end of life" [16,17].

There is consensus about sedatives being used with the intention reducing consciousness, thus improving symptom control. However, it is a controversial issue if terminal sedation should only be used in uncontrollable physical symptoms like pain and dyspnoea or in mental symptoms like fear and depression as well. Empirical works reveal a very heterogeneous practice [18-20].

There are also different attitudes with regard to the depth and manner of the sedation: Some physicians aim to carry out the sedation maintaining consciousness and primarily taking advantage of the anxiolytic effects so that the ability to make contact is impaired as little as possible [10], while others carry out a deeper sedation for more effective symptom control [17,21,22].

Some physicians carry out the sedation until the patient's death, others object to such a continuous sedation and carry out intermittent treatment [10].

Terminal sedation is frequently combined with the withdrawal of life-prolonging measures; however, especially the termination of nutrition and fluid supply under terminal sedation is controversial. Critics regard it as an intentional shortening of life and thus as a form of active euthanasia [3,23,24]; supporters consider the continuation of nutrition and fluid supply under terminal sedation as inconsistent because the dying process is just being extended without any benefit for the patient [25-27].

Against the background of these different attitudes, we interviewed German-speaking medical ethics experts by questionnaire on the term and the moral acceptance of terminal sedation in order to find out how they think about this topic. We were especially interested in whether experts with a professional medical and nursing background think differently about the topic than experts without this background.

We decided to use "terminal sedation" as a point of departure, because in the German-speaking area this is still the more commonly used term: A Google search delivers about 700 hits for the German phrase "terminale Sedierung", but only 226 for "palliative Sedierung", and in the literature database BELIT of the German Reference Centre for Ethics in the Life Sciences [29] eight articles can be found using "terminale Sedierung" in the title, but only one using "palliative Sedierung".

## Methods

Our survey among medical ethics experts was carried out by questionnaire. This consisted of five batteries of questions: In question 1, we asked about the extent of the respondents' previous involvement in the topic "terminal sedation". Questions 2.1 to 2.3 dealt with the definition of the term "terminal sedation" concerning intention, depth of sedation and manner of sedation. In question 3.1, respondents were to specify which term they preferred ("sedation at the end of life", "palliative sedation", "terminal sedation" or "others, please specify:"); question 3.2 offered respondents the opportunity to describe their personal understanding of the various terms in their own words. Question 4 dealt with the moral evaluation of different scenarios. In questions 5.1 to 5.5, we collected various sociodemographic data (gender, age, professional background, religiousness and affiliation to a religious community).

The survey of medical ethics experts took place in June 2005; a recall was carried out in September 2005. The members of the Academy for Ethics in Medicine (AEM) were chosen as test persons. The AEM is the largest scientific expert society for ethics in medicine in the German-language area. Its members are from various special fields, represent various professions and deal with medical ethical issues in science and practice.

All 477 members of the AEM whose place of residence at the time of the survey was in Europe were addressed. 435 lived in Germany, 18 in Austria, 13 in Switzerland and eleven in other European countries.

Calculations were performed using standard statistic software packages (Statistica 5.1, StatSoft Inc, Tulsa, USA and SPSS for Windows 12.0, SPSS Inc., Chicago, Illinois, USA). We tested normal distribution by the Kolmogorov-Smirnov-Test. Wilcoxon-matched-pairtest and Mann-Whitney-U-test were applied to analyse differences between two continuous variables. For dichotomous and categorical data we tested the relationship by entering frequencies into a 2 × 2 table, using the chi square test and Fisher's exact test. Linear regression analysis using the least square method was applied for correlation analysis. For all statistical tests p < 0.05 was considered to be significant.

## Results

### Response rate and sociodemography

281 completed questionnaires were returned, which approximates a response rate of 59%.

The respondents had a professional background in the fields of medicine, nursing care, philosophy, theology, law and/or others. The distribution in percent concerning gender and special fields corresponded with the distribution of the studied members of the AEM (cf. table 1). 18% of the respondents categorised themselves as not or little religious (scale values 1–2), 53% as average religious (scale values 3–8) and 17% as very religious (scale values 9–10). 77% of the respondents were affiliated to a Christian church (Catholic 32%, Protestant 43%) or other religious community.

**Table 1:** Sociodemography of the respondents in comparison to the studied members of the AEM

	Respondents	Members of the AEM
*Gender*		
Men	71%	72%
Women	28%	28%

*Age*		
< 40 years	22%	
41–50 years	26%	(not known)
51–60 years	28%	
> 60 years	23%	

Professional background*		
Medicine	63%	53%
Nursing	6%	5%
Philosophy	27%	19%
Theology	18%	19%
Law	7%	7%
Other	10%	13%

* More than one answer possible

### Previous involvement in the topic

92% of the respondents knew the term "terminal sedation". According to their own estimation, 32% had dealt with the topic in detail, 46% had dealt with it a little. 14% stated to have heard the term but not to have dealt with the topic any further. Only 8% had never heard of the term before. The extent of the previous involvement in the topic did not depend on the respondents' professional background (n.s.).

### On the understanding of the term

In question 2, the medical ethics experts were asked about their understanding of the term "terminal sedation"; for this, two possible answers were provided with regard to intention, depth and manner of sedation.

73% of the respondents would only speak of terminal sedation when sedation until death is intended. 27% would also use the term when the patient is sedated at the time of death, without sedation until death explicitly intended in the first place.

For 45% of the respondents, terminal sedation comprised the complete elimination of consciousness, 55% also used the term for less deeper forms of sedation, in which consciousness is clouded but the patient is still able to have conscious perceptions. Persons with a professional background in medicine or nursing care (n = 202) – in the following summarised as persons with medical background – significantly more frequently preferred the second, broader definition of the term (complete elimination of consciousness: 40%; less deeper sedation: 60%) than persons without a medical background (complete elimination of consciousness: 56%; less deeper sedation: 44%) (p < 0.05).

For 45% of the respondents, continuous sedation until death was a prerequisite for terminal sedation. 54% stated to accept the term even if sedation is interrupted and the patient is awake for some time. Persons without a medical background showed a preference for answer option 1 (continuous sedation: 60%; intermitted sedation: 40%), whereas persons with a medical background showed a preference for answer option 2 (continuous sedation: 38%; intermittent sedation: 62%) (p < 0.05).

### Alternative terms

In question 3, various alternative terms were specified and respondents could opt for the term or terms they preferred. Furthermore, they were given the opportunity to describe their own understanding of the respective terms in form of free text comments.

38% of the respondents stated a preference for the term "sedation at the end of life", 49% came out in favour of the term "palliative sedation" and 37% of the term "terminal sedation". (cf. table 2) Persons with a medical background significantly more frequently preferred the term "palliative sedation" (p < 0.05), whereas persons without a medical background preferred the term "terminal sedation" (p < 0.05). However, previous involvement in the topic did not influence answering behaviour (n.s.).

**Table 2:** Preference for the different terms (more than one answer possible)

	Respondents with medical background	Respondents without medical background
Sedation at the end of life	38%	36%
Palliative sedation	53%	41%
Terminal sedation	32%	46%

The comments on the various terms show that "sedation at the end of life" was regarded as a generic term (n = 31), which was, however, criticised as being little precise (n = 13). The term "palliative sedation" was preferred because it puts the focus on symptom control and reduction of suffering (n = 71) and is also used independent from the end of life (n = 29). "Terminal sedation" was frequently equated with sedation until death (n = 30); some of the respondents regarded the term as unfortunate because it has a negative connotation and is easy to misunderstand as "terminating" (n = 23). For 16 persons, the term "terminal sedation" as an etymological misunderstanding included the deliberate intention to kill.

### Moral evaluation

In question 4, the medical ethics experts were confronted with different scenarios and asked to morally evaluate them (acceptable/not acceptable/don't know). Starting point for all scenarios was the patient to be suffering from a severe disease leading to his death and to be receiving artificial feeding. For the scenarios we used three variables (patient's life expectancy, the source of suffering, and the type of decision), resulting in the following variants:

• "Patient is suffering from a fatal disease and will die soon" (dying patient)

• "Patient is suffering from a fatal disease but will not die in the foreseeable future" (patient with unfavourable prognosis)

• "Patient is suffering from severe pain which cannot be controlled by drugs" (physical suffering)

• "Patient is not suffering from pain but from a severe depression caused by the disease; psychological support and drugs cannot alleviate his suffering" (mental suffering)

• "Patient wants sedation until his natural death occurs" (only sedation)

• "Patient wants withdrawal of nutrition under sedation" (sedation + withdrawal of nutrition).

• "Patient wants withdrawal of nutrition without sedation" (only withdrawal of nutrition)

These variants were combined to twelve scenarios (cf. table 3).

**Table 3:** Moral evaluation of different scenarios (details in valid percent, difference at 100% due to rounding)

**Dying patient**		Acceptable		Not acceptable		Don't know	
		
		M	øM	M	øM	M	øM
Physical suffering	Only sedation	98	97	1	1	1	1
	Sedation + withdrawal of nutrition	86	89	12	8	3	3
	Only withdrawal of nutrition	92	95	6	5	3	0
Physical suffering	Only sedation	61	52	23	39	16	9
	Sedation + withdrawal of nutrition	55	44	29	46	16	10
	Only withdrawal of nutrition	68	63	21	22	11	14

**Patient with unfavourable prognosis**		Acceptable		Not acceptable		Don't know	
		
		M	øM	M	øM	M	øM

Physical suffering	Only sedation	74	66	17	23	9	11
	Sedation + withdrawal of nutrition	63	56	28	30	9	14
	Only withdrawal of nutrition	78	71	17	22	5	7
Mental suffering	Only sedation	37	36	43	47	20	18
	Sedation + withdrawal of nutrition	32	27	48	52	20	21
	Only withdrawal of nutrition	52	50	34	39	14	12

M = respondents with a medical background; øM = respondents without a medical background

Terminal sedation in dying patients with uncontrollable physical symptoms was regarded as morally acceptable by nearly all respondents (98% of respondents with a medical background and 97% of respondents without a medical background; cf. table 3). The simultaneous withdrawal of nutrition was unacceptable for every tenth respondent on average. The acceptance of terminal sedation in dying patients with incurable mental suffering was considerably lower: Sedation alone was acceptable for 61% and 52% respectively; sedation together with withdrawal of treatment was acceptable for 55% and 44% respectively.

Terminal sedation in patients with unfavourable prognosis was considered acceptable by fewer respondents, whereas sedation in patients with physical symptoms was still regarded as acceptable by a (distinct) majority of respondents (without withdrawal of nutrition: 74% and 66% respectively; with withdrawal of nutrition: 63% and 56% respectively). Terminal sedation in patients with unfavourable prognosis with mental suffering, however, met with disapproval rather than acceptance. At the same time it can be noticed, that every fifth respondent chose the answering option "don't know" for the respective scenarios.

When comparing acceptance for the termination of nutrition without sedation with acceptance for withdrawal of treatment under terminal sedation, it becomes obvious that acceptance for withdrawal of treatment without sedation was higher – especially concerning those scenarios in which sedation as such had met with less acceptance (dying patient with mental suffering: +13 and +19 percentage points respectively; patient with unfavourable prognosis with physical suffering: +15 and +15 percentage points respectively; patient with unfavourable prognosis with mental suffering: +20 and +23 percentage points respectively).

Furthermore, a comparison between respondents with or without a medical background reveals that those with a medical background tended more to consider the various scenarios as morally acceptable. This tendency became especially obvious in the scenarios of dying patients with mental suffering (only sedation: +9 percentage points; sedation + withdrawal of nutrition: +11 percentage points) and patients with unfavourable prognosis with physical suffering (only sedation: +8 percentage points; sedation + withdrawal of nutrition: +7 percentage points).

## Discussion

### On representativity

Because of the response rate of 59% and the large degree of accordance between the respondents and the members of the AEM concerning the distribution according to gender and professional background, it can be assumed that our results properly reflect the current state of the debate within the studied society for medical ethics. Representative statements about the attitudes of other persons involved in the medical ethical discourse in the German-language area are, however, not possible.

### No consistent terminology and no consistent definition of the term

The survey confirmed the impression gained from the literature that the terms used in the debate are understood and applied quite differently.

Medical ethics experts with a medical background tended more towards a broader understanding, which focuses on symptom relief: They generally regarded terminal sedation as a palliative medical treatment option, which is used when other measures of symptom control are not sufficient any more, and which is only continued as deep and as long as is necessary for sufficient symptom relief. Such sedation is not necessarily limited to the end of life. Accordingly, they preferred the term "palliative sedation", which better expresses the physician's intention of the sedation measure. Medical ethics experts without a medical background, however, preferred the term "terminal sedation", the majority of them understood it as an elimination of consciousness carried out until the patient's death.

Since the discourse on medical ethics is an interdisciplinary one, these findings have a clear implication for the further debate: in order to meet the danger of using the same terms with different meaning, we regard it as indispensable that those involved in the medical ethical discourse give a definition of the terms they use. In the long run, a more consistent and precise terminology is desirable. We recommend to use the terms "palliative sedation" and "terminal sedation" as synonyms for treatments aiming to control untreatable symptoms in (pre-)terminal patients by reduction of consciousness. The treatment is to be regarded as ultima ratio and normally results in patients loosing their ability to make contact as soon as treatment is started. In all other cases of medically indicated sedation we recommend to speak only of sedation.

### Morally difficult decisions

The survey shows that acceptance of terminal sedation decreases when the patient is not yet dying, when sedation is used because of uncontrollable mental suffering, and when life-sustaining measures are terminated during sedation. Accordingly, terminal sedation in dying patients with physical suffering without withdrawal of treatment met with the highest acceptance, terminal sedation in patients with unfavourable prognosis with mental suffering and simultaneous withdrawal of treatment met with the highest disapproval.

The variant "mental symptoms" (in our scenarios: a severe and treatment refractory depression caused by the disease) had the greatest effect: It resulted in a decrease of the rate of acceptance of up to 45 percentage points in comparison to the respective scenarios in a patient with physical symptoms. At the same time, the number of those who opted for the answer "don't know" rose to 21%. From this it can be inferred that the medical ethics experts consider terminal sedation in mental suffering as a morally difficult decision; however, many of them do not yet have a concluding opinion. These results correspond to the fact that in palliative medical literature mental suffering as an indication for terminal sedation is regarded as difficult [15,20,28]. However, the relatively high consent especially in respondents with medical background (32% to 63%) is rather surprising and should contravene actual practice: even though there are no empirical data available, from their knowledge of treatment of palliative patients in Germany the authors assume that in practice terminal sedation is hardly ever applied in treatment refractory depression.

Furthermore, the fact should be considered that the withdrawal of nutrition under sedation was regarded more morally problematic than the withdrawal of nutrition without sedation. The reason for this could be that in case of sedation the patient concerned is not able to change his decision on the withdrawal of treatment. We consider it the task of future medical ethics research to clarify to what extent the observed difference in value is factually and normatively justified.

## Conclusion

Our survey showed that for the studied medical ethics experts terminal sedation involves considerable moral uncertainties. This results in a further need for medical and medical ethics research and discussion – especially concerning the medical indication and moral evaluation of terminal sedation in patients with uncontrollable mental suffering as well as the withdrawal of treatment under sedation measures. The way we see it, a more precise terminology, reflecting the circumstances of sedation, is the prerequisite for an appropriate ethical debate.

## Competing interests

The author(s) declare that they have no competing interests.

## Authors' contributions

AS, MK, and DB developed the questionnaire and carried out the survey. JH gave methodological advices and performed the statistical analysis. MK and DB drafted the section "Background", JH the section "Methods", and AS the sections "Result", "Discussion", and "Conclusions". All authors read and approved the final manuscript.

## Pre-publication history

The pre-publication history for this paper can be accessed here:


